# Atomic layer deposited TiO_2_ nanofilm on titanium implant for reduced the release of particles

**DOI:** 10.3389/fbioe.2024.1346404

**Published:** 2024-04-26

**Authors:** Xiangyu Zhao, Xiaoxuan Zhang, Zilan Zhou, Fanchun Meng, Ruilin Liu, Mengyuan Zhang, Yujia Hao, Qingpeng Xie, Xiaojun Sun, Bin Zhang, Xing Wang

**Affiliations:** ^1^ Shanxi Medical University School and Hospital of Stomatology, Taiyuan, China; ^2^ State Key Laboratory of Coal Conversion, Institute of Coal Chemistry, Chinese Academy of Sciences, Taiyuan, China; ^3^ Department of Stomatology, The First Hospital of Shanxi Medical University, Taiyuan, China

**Keywords:** atomic layer deposition, titanium dioxide, titanium implant, particles release, friction and corrosion

## Abstract

**Objective:** Titanium implants are widely used in surgeries for their biocompatibility and mechanical properties. However, excessive titanium particle release can cause implant failure. This study explores Atomic Layer Deposition (ALD) to coat commercially pure titanium (Cp-Ti) with TiO_2_, aiming to improve its frictional and corrosion resistance while reducing particle release. By comparing TiO_2_ films with varying ALD cycle numbers, we assess surface properties, particle release, friction, and corrosion performance, providing insights into mitigating particle release from implants.

**Methods:** Cp-Ti surfaces were prepared and coated with TiO_2_ films of 100, 300, and 500 ALD cycles. Surface characterization involved SEM, EDX, and XRD. Friction was tested using SEM, nanoindentation, and ICP-MS. Corrosion resistance was evaluated through immersion tests and electrochemical analysis. Cytotoxicity was assessed using BMSCs.

**Results:** Surface characterization revealed smoother surfaces with increased ALD cycles, confirming successful TiO_2_ deposition. Friction testing showed reduced friction coefficients with higher ALD cycles, supported by nanoindentation results. Corrosion resistance improved with increasing ALD cycles, as evidenced by electrochemical tests and reduced titanium release. Cytotoxicity studies showed no significant cytotoxic effects.

**Conclusion:** ALD-coated TiO_2_ films significantly enhance frictional and corrosion resistance of titanium implants while reducing particle release. The study underscores the importance of ALD cycle numbers in optimizing film performance, offering insights for designing implants with improved properties.

## 1 Introduction

Commercially pure titanium (Cp-Ti) has been acknowledged as a premier implant material with bio-safety and nearly 1,000 tons of titanium is used annually in clinical applications in diverse applications, such as artificial joints, dental implants and heart valves (Xu et al., 2020). However, despite the excellent performance, many cases of implant Ti failure still exist. The failure rate of dental implants has been reported to be about 1%–20% (Alves et al., 2017), and more than 35% for orthopedic implants (Tobin, 2017). In 75% of these cases, implant failure was due to aseptic loosening and impaired implant fixation (Awad et al., 2022). It has been shown that titanium implants in bone can continuously release titanium species (He et al., 2016), and the release of titanium ions and titanium particles can contribute to peri-implantitis and aseptic implant loosening (Eger et al., 2017). The released Ti species are not confined to the periphery of the implant, and even migrate with the blood and progressively accumulate in distal organs (Heringa et al., 2018), which leads to systemic hypersensitivity and allergic reactions. Released Ti particles are not limited to the periphery of the implant; they can migrate with the bloodstream and gradually accumulate in distant organs. In most studies, Ti nanoparticles have been found to cause oxidative stress, tissue pathology changes, carcinogenesis, genetic toxicity, and immune disruption (Shakeel et al., 2016). Worryingly despite the reported importance of excessive titanium species release on implant success and systemic health, the problem is routinely ignored by clinicians, patients and implant manufacturers, lacking sufficient attention and preventive measures.

Mechanical friction and corrosion are the main reasons for the physical degradation of titanium implants (Mombelli et al., 2018). Mechanical friction and corrosion are the primary factors leading to the degradation of titanium implants. Even within the bone-encased portions of the implant, frictional corrosion still occurs. This phenomenon arises from minute relative vibrations between two surfaces (implant surface and bone surface) under mechanical loads (friction), leading to irreversible corrosion degradation of the implant through electrochemical interactions with the surrounding environment (Berbel et al., 2019; Prestat and Thierry, 2021). Generally, increasing the strength of Ti and Ti alloys against friction and corrosion suppresses the mechanical friction to titanium particles. On the other hand, corrosion originates from the surface chemical reaction of metallic Ti in acidic environments. Although TiO_2_ films formed spontaneously on Ti surfaces in contact with oxygen are resistant to corrosion when in contact with acids and reactive substances (Peron et al., 2021). However, the growth of the oxide layer is a dynamic equilibrium process involving oxidation and reduction reactions. Under certain conditions, the growth rate of the TiO_2_ layer may be slow or non-uniform, resulting in an unstable layer quality that is easily damaged by external factors (Lara Rodriguez et al., 2014).

Surface overcoating has attracted extensive interest from researchers in recent years due to its ability to maintain the surface morphology of the implant and to be matched to the mechanical properties. There are various coating techniques available for implant modification. Such as anodic oxidation (Fazel et al., 2018), pulsed laser deposition (PLD) (Gnanavel et al., 2018), and physical vapor deposition (PVD) (Esmaeili et al., 2017), have been implemented to improve corrosion resistance. Forming anodic oxide coatings requires precise control of multiple process parameters such as electrolyte composition, temperature, and current density (Leinenbach and Eifler, 2009) and, therefore, requires complex process control and operation techniques with poor reliability and stability. The PLD technique is subject to effects such as phase explosion, which causes large particle sputtering, thus reducing the film quality and generating localized high-temperature effects on the titanium surface, which may lead to lattice structure alterations and thermal stresses in the titanium material, which may impact the properties and structure of the material (Deng et al., 2021). In addition, the coating thickness of PVD deposition is inhomogeneous on the surface of complex-shaped implants (Mohseni et al., 2014). This could lead to delamination of the substrate with the thinner coating, and the local oxide film will break if a critical strain is reached. It is still necessary to develop implant modification technique that can achieve uniform deposition at low temperatures and is easy to handle.

Atomic layer deposition (ALD) has unique advantages in sub-nanometer film thickness and uniformity control with increasing applications in biomedical advanced materials (Yang, 2020). Atomic Layer Deposition (ALD) is a precise thin-film deposition technique that enables highly accurate control over film thickness and composition by depositing films atom by atom on the material surface. During the reaction process, precursor molecules adhere to the substrate surface in a “self-limiting” manner, forming a single-molecule layer. Once the substrate surface is fully covered, excess precursor molecules no longer adsorb, resulting in the automatic termination of the deposition process. This means that regardless of the quantity of precursor molecules provided, only enough molecules can adsorb and participate in the reaction. This ensures the uniformity and consistency of the film. Compared to PCD, ALD is based on surface self-limiting and self-saturating adsorption reactions (Kim et al., 2023), which enables precise deposition at the atomic level on the complex surface of implants (Blendinger et al., 2021), and one study reported that the thickness of the film deposited per cycle on the implant surface is only 0.097 nm (Yang et al., 2017). The uniformity and consistency of ALD film avoid problems such as surface inhomogeneity and thickness variation. Compared with PLD, which usually operates at 500°C–800°C, ALD can even be conducted at a low temperature of 100°C (Wree et al., 2023), avoiding heat damage and shape changes to the implant material and maintaining the integrity and structural stability of the implant. The homogeneous films deposited by ALD are stable in properties; consequently, ALD-deposited TiO2 films have the potential to be used for implant surface coatings (Hashemi Astaneh et al., 2021).

**FIGURE 1:**
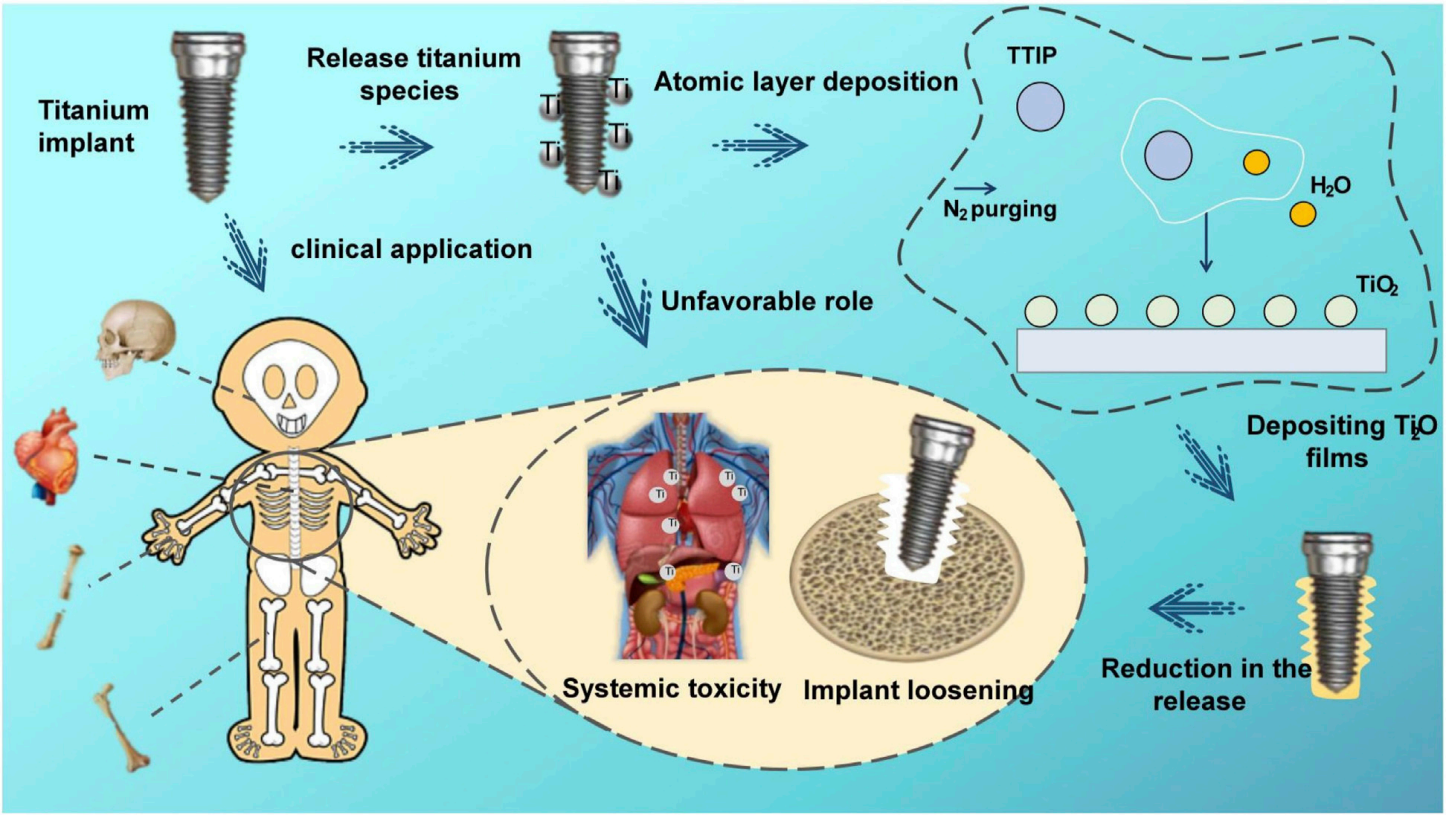
Schematic illustration of the research of TiO_2_ films deposited through ALD on CP-Ti.

Herein, we have used ALD technology to overcoat the titanium surface with stable TiO_2_ film to enhance its frictional corrosion resistance and reduce the release of titanium particles (Figure 1). To our knowledge, this is the first study of the utilization of ALD TiO_2_ films to solve the problem of titanium particle release from implants. The simplicity of the ALD technique, the lower working temperature, and the uniformity and stability of the films compared to other deposition techniques make it a viable solution. In addition, to further evaluate the friction and corrosion resistance of the films with different cycles and the particle release after friction and corrosion, we deposited 100, 300, and 500 cycles of TiO_2_ films on Cp-Ti using the ALD technique. Through this study, we intended to provide a feasible solution to the problem of implant particle release, demonstrating that the ALD modification technique has tremendous potential in implant research to enhance the performance and long-term success of titanium implants, providing a new direction and opportunity for research to reduce titanium particle release from implants.

## 2 Materials and methods

### 2.1 Sample preparation and thin film deposition

A commercial pure Ti (CP-Ti) plate was utilized as a surrogate for titanium implants in this study. CP-Ti (10 mm × 10 mm × 5 mm) was ground sequentially with silicon carbide sandpaper of different particle sizes (600, 800 and 1200), and then cleaned sequentially with acetone and ethanol by ultrasonication for 15 min. The ALD process was carried out in a homemade closed-chamber type ALD reactor. High-purity N_2_ (99.999%) was utilized as a carrier gas.

The selection of Titanium tetraisopropoxide (TTIP) and ultrapure water as precursors offers advantages such as usability, affordability, chemical stability, availability, low maintenance, safety, and a lower deposition temperature range (150°C–300°C) (Niemelä et al., 2017). Deposition at 160°C prevents thermal damage to the substrate material at high temperatures, which holds significant importance for the structure and applications of titanium implants. TTIP and water were kept at 80°C and 25°C, respectively. The pulse, exposure, and purge times for titanium tetraisopropoxide were 1, 8, and 20 s, and those for H_2_O were 0.1, 8, and 25 s, respectively. To explore the role of different number of cycles on particle release, we deposited 100, 300, and 500 cycles of ALD layers.

### 2.2 Surface characterization

Scanning Electron Microscopy (SEM) using the JSM-7900F was employed to meticulously observe the surface morphology of thin films, providing crucial insights into the microstructure and surface features of different cyclically deposited films. Additionally, Focused Ion Beam (FIB) was used to cut samples for Transmission Electron Microscopy (TEM), enabling the measurement of nanofilm thickness and cross-sectional elemental distribution.

Energy-Dispersive X-ray Spectroscopy (EDX) was utilized to quantitatively analyze the elemental composition of the films, helping determine whether variations in titanium-to-oxygen ratios exist across different cycles. The combination of SEM, FIB-TEM, and EDX provides a comprehensive understanding of the morphology and elemental distribution of TiO_2_ coatings.

Research findings indicate that rutile-type TiO_2_ nanoparticles cause more significant damage to murine bone tissue compared to anatase-type TiO_2_ nanoparticles (Cheng et al., 2021). By comparing measured XRD patterns with standard reference cards in databases, the phase structure can be accurately determined. Therefore, we employed a PANalytical X’Pert Pro X-ray Diffractometer (XRD) to examine the film’s structure within an angular range of 20°–60°.

### 2.3 Friction tests

In the oral environment, dental implants are exposed to various components present in saliva, including organic substances, dissolved oxygen, inorganic anions (such as Cl^−^, HPO_4_^2-^, HCO^3-^), and cations (such as Na^+^, K^+^, Ca^2+^, Mg^2+^), as well as amino acids and proteins. These constituents may contribute to the degradation of the TiO_2_ layer (Ossowska and Zieliński, 2020). To study oral implants, we conducted friction-corrosion tests simulating the oral microenvironment, also known as bio-tribocorrosion. In these experiments, we selected artificial saliva as the medium to better understand the performance and durability of titanium-based implants and prostheses in the oral environment.

According to ASTM G119-93 (ASTM, 1993), a material surface property tester (MFT-4000, Huahui, China) was used to perform the friction corrosion test under artificial saliva immersion. In this study, GCr15 steel balls were used as the friction counterpart because the hardness of GCr15 bearing steel can reach HRC 58–63 after heat treatment, which is much higher than that of titanium alloy (HRC 28–33) which would not cause plastic deformation of the friction counterpart leading to wear and thus affect the experimental results during the tribocorrosion experiments (Zhou et al., 2019). A normal force of 5 N was applied to the counterpart, using the Hertzian contact stress model, corresponding to a maximum contact pressure of about 950 MPa, while the radius of the wear track was set to 5 mm, the speed of the sample table was set to 200 mm/min, and the test time was 10 min.

Two different methods were employed to assess the frictional performance. Scratch experiments were conducted using an ultra-nanoindenter with a diamond probe featuring a radius of 1 µm and an angle of 60°. The experiments were performed under a constant load of 500 μN, with a scratch distance of 10 µm and a duration of 1 min.

The surfaces of the four sample groups after friction were examined for surface scratches and particle detachment using SEM. In the presence of titanium nanoparticles in the sample, the distribution of elements no longer exhibits uniformity but appears as discrete atomic clusters. This non-uniformity hinders accurate quantitative analysis. Addressing this challenge, we employed the high-sensitivity ICP-MS (Inductively Coupled Plasma Mass Spectrometry) technique, specifically designed for elemental analysis in samples (Laborda et al., 2014). In this experiment, we collected titanium nanoparticles from artificial saliva dissolved in nitric acid and utilized ICP-MS for precise quantitative and statistical analysis of titanium content in the solution. To gain a more detailed insight into the morphology and size of titanium particles, we further employed SEM.

### 2.4 Corrosion tests

#### 2.4.1 Corrosion resistance test

Infection, medication, dietary factors, periodontal disease, smoking, and systemic illnesses can potentially decrease the pH of normal saliva from 6.3–7.0 to <6.0 (Licausi et al., 2013). Under acidic conditions, the oxide layer on implant surfaces may be compromised, resulting in surface erosion and eventual release of titanium particles in the absence of wear. Commonly used oral medications, such as citric acid, tetracycline, and sodium fluoride, can lower the solution’s pH to <3 (Wheelis et al., 2016), causing rapid breakdown of the implant surface oxide layer and release of Ti particles. Therefore, this experiment simulated different acidic environments in the oral cavity using artificial saliva with pH values of 2.5, 5.5, and 7.0.

Each set of samples was immersed in 2 mL artificial saliva with different pH values, at 37°C for 7 days. SEM was utilized to compare the corrosion status of the samples before and after immersion, and EDX was employed to detect elemental changes on the corroded surface. To assess titanium release in highly acidic conditions, artificial saliva with a pH of 2.5 was collected, and the titanium content was quantified using ICP-MS, followed by statistical analysis. TEM was employed to investigate the morphology and size of particles that might exist in the solution.

#### 2.4.2 Electrochemical tests

The electrochemical workstation (CHI6600E, Chinstruments, China) was used to perform the electrochemical tests on four sets of samples, respectively. A three-electrode setup was used, with the sample as the working electrode, the saturated glyceryl electrode as the reference electrode, and the platinum plate electrode as the counter electrode. Artificial saliva at pH 6.5°C and 37°C was used as the electrolyte. All tests were performed in a simulated oral environment at 37°C ± 1°C. Electrochemical tests were performed according to ASTM-G61 standard. The first step was system stabilization, and after 3600s of open circuit potential (OCP) monitoring, dynamic potential polarization tests and electrochemical impedance spectroscopy (EIS) tests were performed at a stable OCP. EIS tests were performed in the frequency range of 100 kHz-0.005 Hz, and Nyquist plots were plotted using EIS data. The dynamic potential polarization test was performed at a scan rate of 0.5 mV/s, and the corrosion potential (Ecorr) and the corresponding current density (Icorr) were estimated using Tafel curves. The measurements were repeated three times for each condition.

### 2.5 Cytotoxic studies

#### 2.5.1 Cell cultures

Biocompatibility tests were performed according to ISO 7405–2018 (ISO, 2018). The cells used in this test were bone marrow mesenchymal stem cells (BMSCs)Cultivate under conditions of 37°C, 5% CO_2_, and saturated humidity until cells reach 70%–80% growth. Perform passage using 0.25% trypsin. Cells cultivated up until passage 3 (P3) then ready for experimental use. The titanium samples were placed in complete medium and in a 37°C for 72 h to obtain the extracts for the cell experiments.

#### 2.5.2 Fluorescent staining

Cells at a density of 1×10^5^ cells/mL were incubated in confocal dishes for 24 h. Cells were seen to adhere to the wall under an inverted microscope, the original culture medium was discarded, an immersion fluid exchange was performed, and the plates were incubated in an incubator for 24 h. And then rinsed three times with PBS. Add 150 μL of staining working solution A, incubate for 30 min at 37°C protected from light, rinse three times with PBS, add 150 μL of staining working solution B, and incubate for 20 min at 37°C protected from light. Observe the cell morphology at 488 nm under a laser confocal microscope (FV3000, Olympus, Japan).

#### 2.5.3 LDH tests

The cytotoxicity of the samples was assessed by quantifying the release of lactate dehydrogenase (LDH) from BMSCs. Following cultivation, cells were seeded into a 96-well plate and treated with different material extracts. After 12 h of incubation, LDH release reagent was added, and the cells were incubated for an additional hour. The cell culture plate was then centrifuged at 400g for 5 min using a multi-well plate centrifuge. Supernatant (120 µL) from each well was transferred to a new 96-well plate, and absorbance was measured at 490 nm.

### 2.6 Statistical analysis

OriginPro 8.5 and Graphpad Prism9 software have been used for all graphical and statistical analyses. All experiments were conducted three times, except for the LDH assay, which was performed four times. All repeated measurements were expressed as standard deviation (SD). The statistical significance of the data was estimated using a one-way analysis of variance (ANOVA). *p* < 0.05 was considered statistically significant for significance levels, * for *p* < 0.05 and ** for *p* < 0.01.

## 3 Results

### 3.1 Surface characterization

**FIGURE 2:**
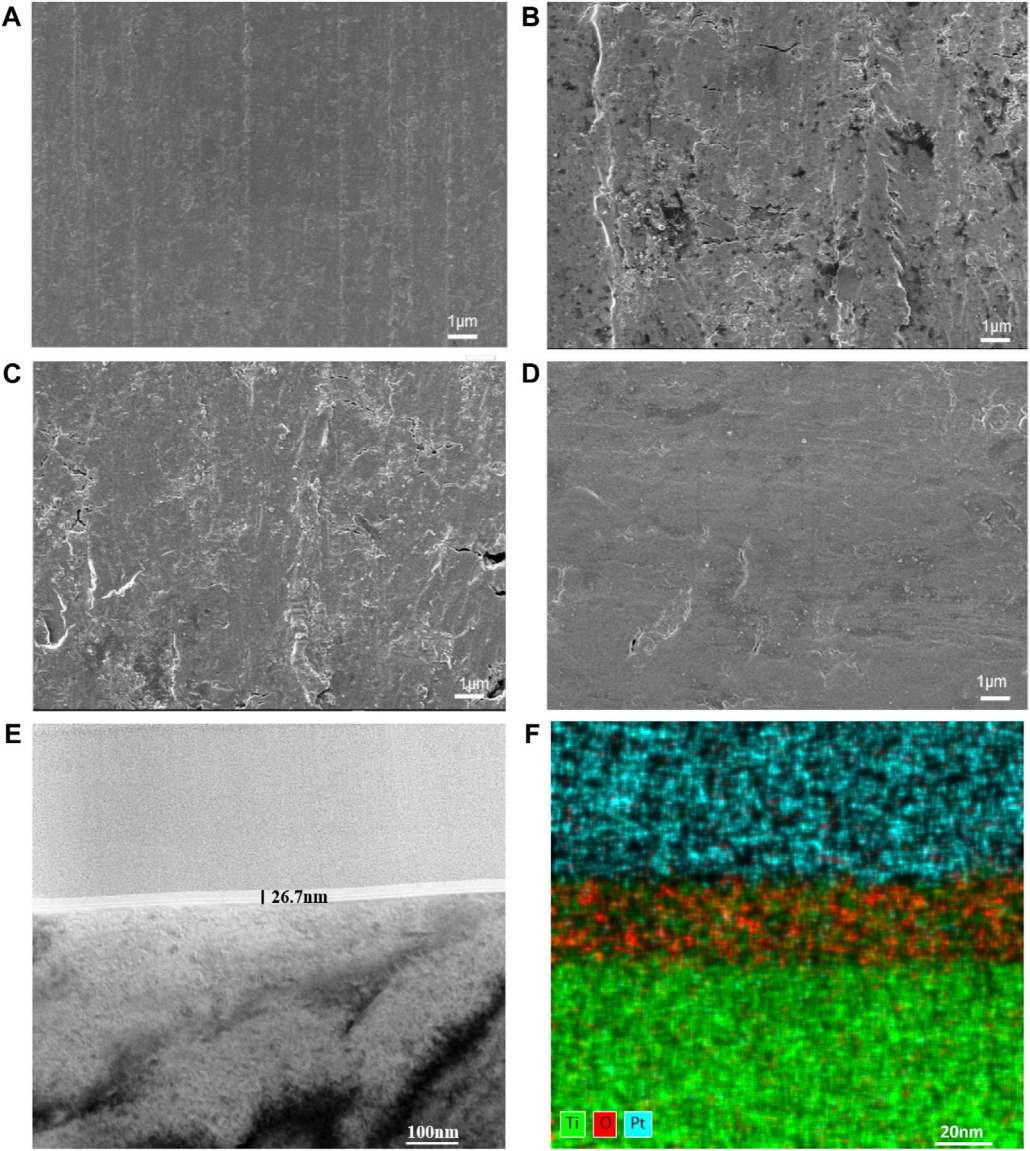
Surface morphologies of CP-Ti with different ALD cycles. **(A)** CP-Ti, **(B)** 100ALD-Ti, **(C)** 300ALD-Ti and **(D)** 500ALD-Ti of SEM. **(E)** TEM and **(F)** mapping for 300ALD-Ti.

The surface morphologies of the modified samples are shown in Figure 2. The SEM results (Figures 2A–D) showed that the surface of the TiO_2_ films generated by ALD was significantly smoother as the number of cycles increased. Since the thinness of TiO_2_ nanofilm increases with the ALD cycle number, a thick enough TiO_2_ film improves the surface smoothness. TEM was employed to examine the 300-cycle group, and the results are depicted in Figures 2E, F. The nanofilm exhibited a thickness of 26.7 nm, with an average TiO_2_ film thickness of 0.089 nm per cycle. Figure 2F illustrates the even distribution of titanium and oxygen elements across the cross-section of the film. The upper layer, containing platinum for detection purposes, is clearly distinguished from the lower layer, representing the pure titanium substrate without additional elements.

**FIGURE 3:**
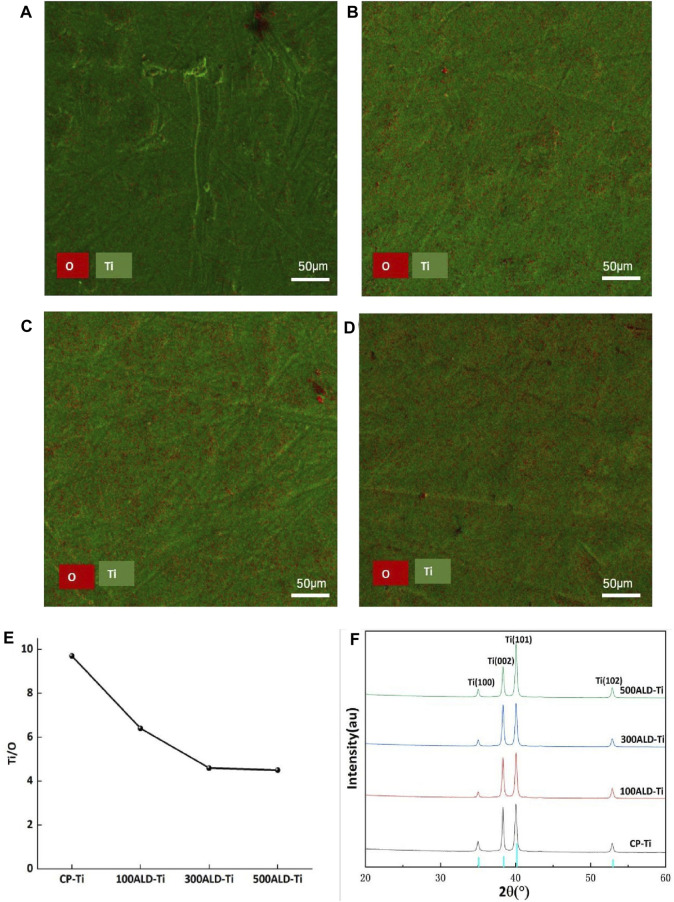
Phase composition and elemental distribution. **(A)** CP-Ti, **(B)** 100ALD-Ti, **(C)** 300ALD-Ti and **(D)** 500ALD-Ti is the mapping diagram after EDX, with O in red and Ti in green. **(E)** Titanium oxygen ratio by EDX spectra. **(F)** XRD patterns. The blue color of the horizontal axis indicates Amorphous TiO_2_ type.

The EDX spectra (Figures 3A–D) show the elemental distribution of titanium and oxygen on the surfaces of each group. Table 1 and Figure 3E indicates the titanium and oxygen content of the sample surface. The increase of oxygen ratio on the surface suggests the covering of TiO_2_ on the Ti surface. Since the oxygen content is not changed after 300 cycles of TiO_2_, a uniform overcoating is realized. The XRD spectra of the samples are in Figure 3F. All diffraction peaks are in good agreement with the standard JCPDS data. The anatase TiO_2_ films had strong diffraction peaks at 2θ = 25.4° and 48.0°, while none of the three groups of ALD-Ti saw this diffraction peak, indicating that the increase in the number of cycles at the deposition temperature of 160°C did not change the crystalline shape of TiO_2_.

**TABLE 1:** Surface analysis of the elemental composition of TiO_2_ films deposited by EDX.

	CP-Ti	100ALD-Ti	300ALD-Ti	500ALD-Ti
Ti (%)	90.7	86.6	82.3	82.0
O (%)	9.3	13.4	17.7	18.0
Ti/O	9.7	6.4	4.6	4.5

### 3.2 Friction tests

**FIGURE 4:**
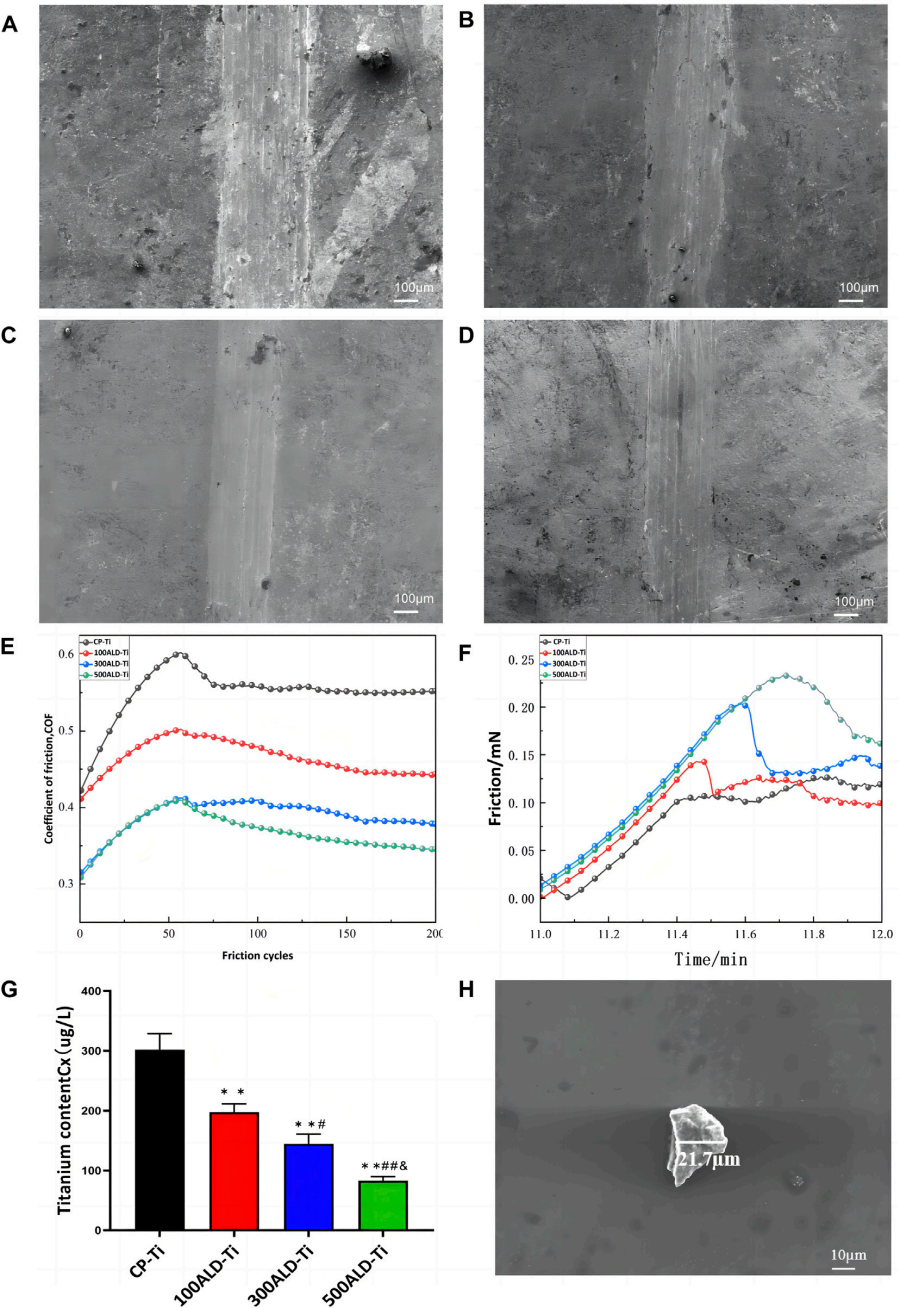
Friction performance. SEM images of **(A)** CP-Ti, **(B)** 100ALD-Ti, **(C)** 300ALD-Ti and **(D)** 500ALD-Ti. **(E)** Influence of different deposition cycles on coefficient of friction. **(F)** Friction analysis of nano scratch testing. **(G)** ICP-MS of post-friction solutions. The results represent the average of three experiments. (n = 3, *p < 0.05, **p < 0.001 compared with the CP-Ti group; #*p* < 0.05, ##*p* < 0.001 compared with the 100ALD-Ti group; and *p* < 0.001 compared with the 300ALD-Ti group). **(H)** SEM of particles in solution.

The surface morphology of the sample after friction is shown in Figures 4A–D. It is evident that all four groups showed friction marks on the surface after friction. However, as the number of cycles increased, the friction marks became shallower, with the 500 ALD-Ti exhibiting the shallowest marks among the four groups.

To facilitate the observation of trends in friction resistance, the friction tests data were smoothed and then plotted, and the relationship between the number of cycles and the coefficient of friction (COF) is illustrated in Figure 4E. Evidently, both the experimental and control groups displayed a progressive increase in friction coefficient followed by a tendency to stabilize. This pattern arises from initial challenges like surface roughness and uneven contact, resulting in a COF increase. As the friction surface adapts and readjusts, the COF gradually stabilizes. And COF is inversely proportional to the friction resistance (Matijošius et al., 2020), from the smooth segment in the graph. As the cycle increases, COF becomes smaller, indicating that the friction resistance increases. The nanoscratch results, as shown in Figure 4F, indicate that the titanium dioxide nanofilm enhances the friction performance, with an improvement observed as the number of cycles increases.

To quantify the titanium species shed from the sample surface into the solution, the solution was acid dissolved and subjected to ICP-MS. The test results are summarized in Figure 4G. Analysis of the data showed that the release of titanium particles was significantly reduced in each experimental group compared to the control group (*p* < 0.001), indicating that the ALD-deposited TiO_2_ coating was able to substantially reduce the release of titanium particles from the implant surface. The reduction in titanium particle release with increasing number of cycles was also observed between the experimental groups (*p* < 0.05), which suggests that the improvement of titanium particle release by the coating is closely related to the number of ALD cycles. SEM (Figure 4H) was employed to determine the size of residual particles in the immersion liquid. A total of 20 particles were identified and measured in the liquid. The particle sizes were predominantly in the micrometer range, exhibiting diverse shapes and forms.

### 3.3 Corrosion tests

#### 3.3.1 Corrosion resistance test

**FIGURE 5:**
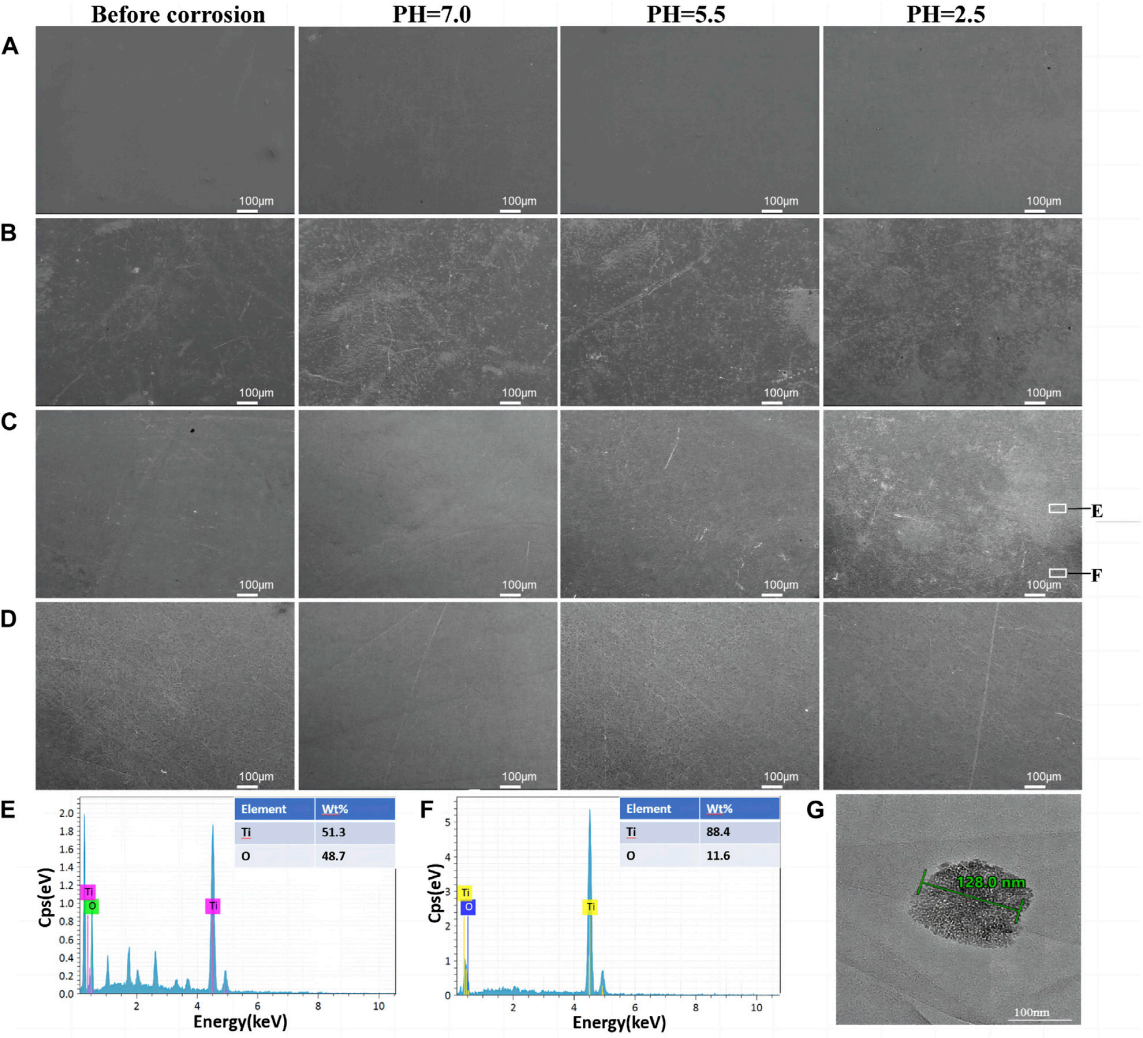
Surface morphology after corrosion. SEM images of **(A)**CP-Ti, **(B)** 100ALD-Ti, **(C)** 300ALD-Ti and **(D)** 500ALD-Ti. **(E,F)** correspond to the EDX spectra of the white boxed region in the last image **(C)**. **(G)** TEM image of Ti particles.

Figures 5A–D show the SEM comparisons before and after corrosion. As can be seen from the figures, there is no change in the four groups of samples before and after corrosion at a PH of 7.0 and 5.5. 100ALD-Ti corrosion traces are more obvious at a PH of 2.5, followed by the 300ALD-Ti. While the 500ALD-Ti and CP-Ti groups had almost no corrosion. This indicates that the corrosion resistance increases with the increase in the number of ALD cycles. The basically unchanged morphology of the CP-Ti before and after corrosion is due to the uniform distribution of the CP-Ti surface and the consistent corrosion resistance, which results in no significant change in the overall surface structure before and after corrosion.

Further EDX analysis of the corroded samples shows that the darker areas in Figure 5C image have a low titanium-to-oxygen ratio (Figure 5E), which corresponds to the TiO_2_ coating. While the brighter region has a high titanium-to-oxygen ratio (Figure 5F), which corresponds to a pure titanium substrate. This suggests that when the titanium implant comes into contact with an acidic environment, the TiO_2_ coating is the first to be corroded, providing protection for the underlying titanium. In addition, as the number of ALD cycles increases, the coatings become thicker, exhibit better corrosion resistance, and provide stronger protection for the titanium. The TEM results (Figure 5G) revealed the presence of nanoscale titanium particles in the solution, exhibiting diverse morphologies and shapes.

**FIGURE 6:**
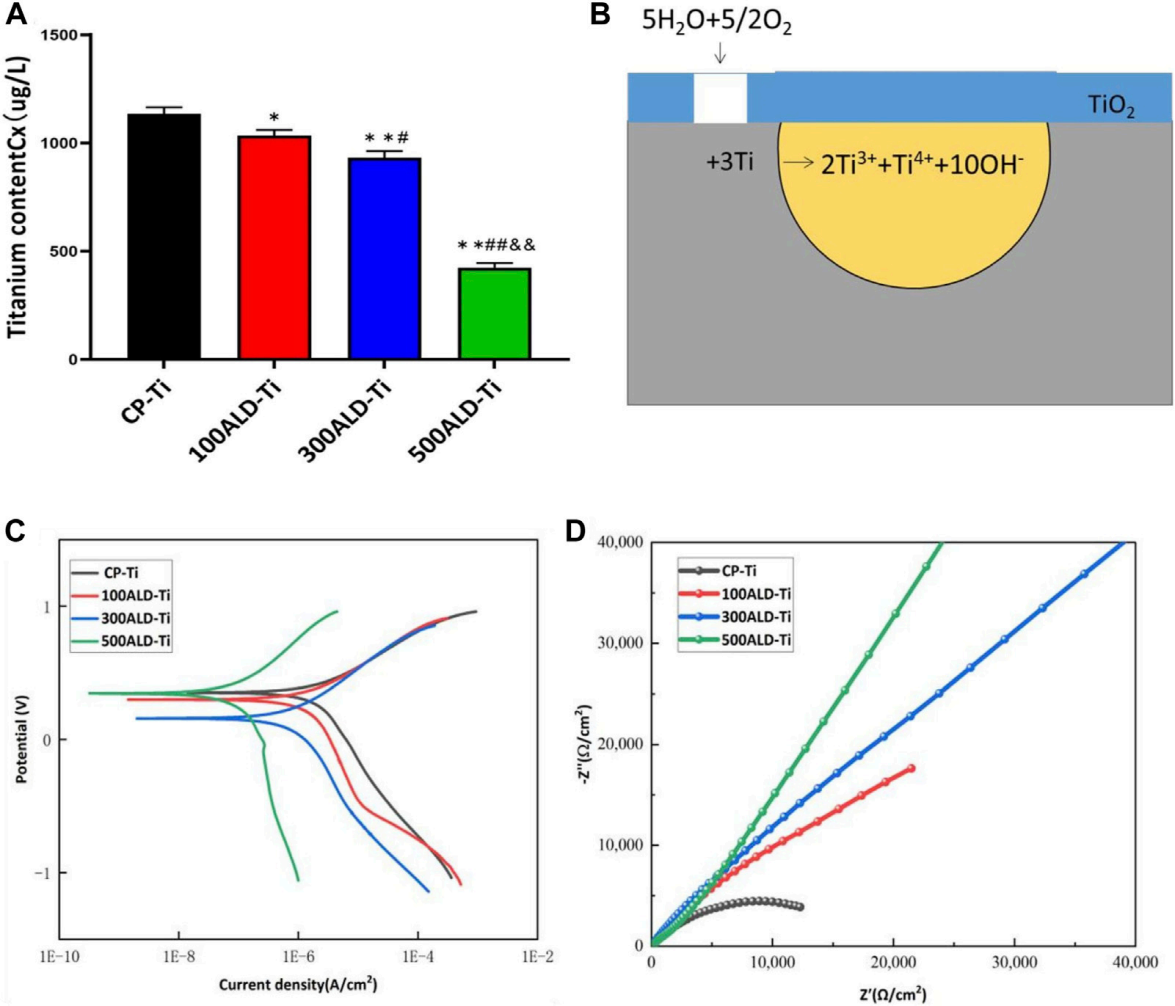
Corrosion performance. **(A)** ICP-MS of post-corrosion solution. The results represent the average of three experiments. (n = 3, *p < 0.05, **p < 0.001 compared with the CP-Ti group; #*p* < 0.05, ##*p* < 0.001 compared with the 100ALD-Ti group; and&*p* < 0.001 compared with the 300ALD-Ti group). **(B)** The reaction route for titanium corrosion. **(C)** Potentiodynamic polarization (Tafel) plots and **(D)** Nyquist plots for electrochemical corrosion.

To determine the titanium ions released from the sample surface into the solution, artificial saliva at a pH of 2.5 was collected and subjected to ICP-MS testing. The results, shown in Figure 6A, revealed that compared to the control group, all experimental groups exhibited a decrease in titanium content (*p* < 0.05). However, in comparison to the other two sample groups, the 500ALD-Ti exhibited a significant reduction in titanium ion release (*p* < 0.001). This finding is consistent with the SEM images, indicating that the decrease in titanium content in the experimental groups may be attributed to the protective effect of the TiO_2_ coating on the underlying titanium substrate. The 500ALD-Ti, which had the thickest deposited TiO_2_ coating, demonstrated the strongest protective effect on the substrate, resulting in the least amount of titanium ion release.

#### 3.3.2 Electrochemical tests

Figure 6B depicts the typical electrochemical reactions that may occur during the corrosion process of uncoated titanium implants without protective coatings. In this process, water molecules play a role in providing hydroxide ions (OH^−^) while oxygen acts as the oxidizing agent. In such a corrosive environment, titanium metal is oxidized into various oxidation states, releasing electrons. It is essential to note that this is just a simplified electrochemical reaction equation representing one possible aspect of the complex process of biocorrosion. The actual scenario is influenced by various factors such as the composition of oral fluids, temperature, pH levels, among others. Therefore, this simplified reaction equation only represents a potential aspect of the corrosion process.

Figure 6C reports the dynamic potential polarization curves of the experimental and control groups. The corrosion potentials of the samples in the experimental group were all shifted to the left compared to the control group. This may indicate a positive effect of the coating on the corrosion resistance of CP-Ti. According to the Tafel equation, the average values of corrosion potential (Ecorr) and corrosion current density (Icorr) are shown in Table 2. The lower the corrosion current density, the lower the corrosion rate. The higher the corrosion potential in the case of similar currents, the lower the corrosion tendency, it can be observed that the corrosion current of experimental group are lower than the control group, indicating that the presence of TiO_2_ coating increases the corrosion resistance of the material. And the number of cycles increased corrosion resistance also increased, 500ALD-Ti shows the best ability of corrosion resistance.

**TABLE 2:** Means and standard deviations of Icorr and Ecorr for each group, n = 3.

	CP-Ti	100ALD-Ti	300ALD-Ti	500ALD-Ti
Icorr (A/cm^2^)	(3.44 ± 2.29)	(1.41 ± 0.62)	(7.17 ± 2.25)	(1.46 ± 1.59)
	E−6	E−6	E−7	E−7
Ecorr(V)	0.14 ± 0.04	0.21 ± 0.12	0.22 ± 0.08	0.24 ± 0.07

Figure 6D reveals the Nyquist (real vs. imaginary impedance) plots used to study the EIS data. The Nyquist plot reveals a significant increase in capacitance loop for the experimental group compared to the control group. A larger capacitance loop diameter correlates with higher corrosion resistance. This indicates that the 500ALD-Ti coating has the highest corrosion resistance and is considerably higher than the control group. 100ALD-Ti has the smallest semicircular ring and therefore has the lowest corrosion resistance in the experimental group. However, 100ALD-Ti still has corrosion resistance compared to the control group.

### 3.4 Cytotoxic studies

**FIGURE 7:**
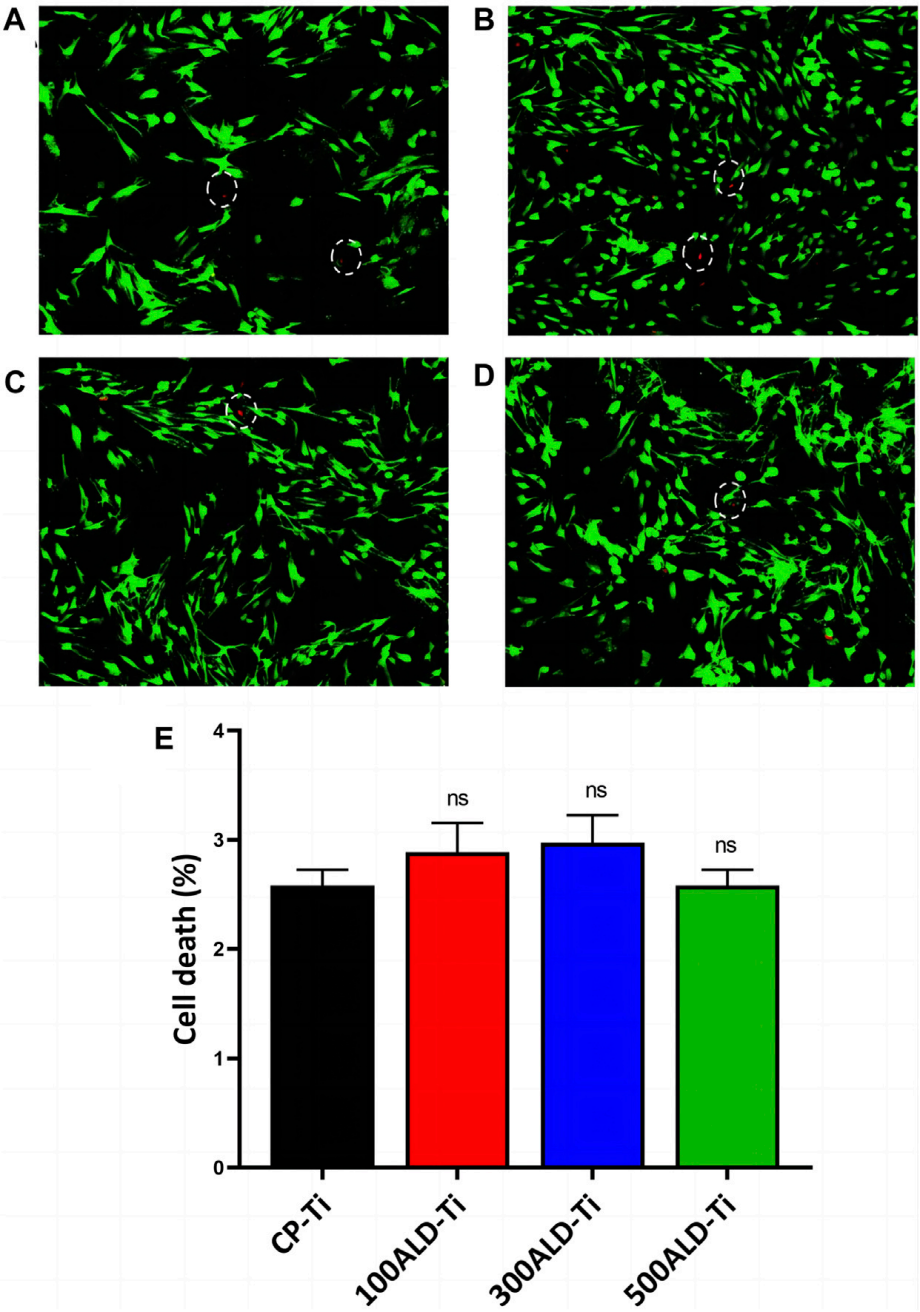
Cytotoxic assays. Fluorescence images after live/dead staining of bone marrow mesenchymal stem cells (BMSC) after culturing for 48 h **(A)** CP-Ti **(B)** 100ALD-Ti **(C)** 300ALD-Ti and **(D)** 500ALD-Ti. **(E)** LDH assay of BMSC. The results represent the average of three experiments. (n = 4, ns mean *p* > 0.05).

Because BMSCs are sensitive to external environmental changes and materials, and can reflect the effect of implant modification materials on cell activity and function, BMSCs are involved in the bone tissue regeneration and repair process *in vivo*, which is important for implant compatibility and bioactivity, BMSCs were selected for this test. To analyze the cytotoxicity, confocal microscopy experiments were performed using both xanthophyll-AM and propidium iodide (PI) solutions, which were used to stain live and dead cells, respectively, and the results are displayed in Figures 7A–D. There were fewer dead cells in both the control and experimental groups.

In LDH test on BMSCs indicated that both pure titanium and ALD-deposited TiO_2_ films were not cytotoxic and were not correlated with the number of cycles. The results of the cytotoxicity test are shown in Figure 7E. Compared to the CP-Ti group, the cell death rates in all other groups did not show a statistically significant increase.

## 4 Discussion

Implant surface degradation in the human body is caused by both friction (resulting in mechanical particle breakdown) and corrosion (leading to chemical degradation of soluble metal ions) (Delgado-Ruiz and Romanos, 2018). Titanium implants with TiO_2_ coatings exhibit strong resistance to friction and corrosion (Matijošius et al., 2020). To address the issue of particle release due to friction and corrosion of these implants, this study deposited thin TiO_2_ films using the ALD technique to create protective layers. ALD’s step-by-step deposition process allows precise control at the atomic level, ensuring uniformity and accurate thickness control of the film (Kim et al., 2021).

The crystalline shape of TiO_2_ deposited by ALD is related to the deposition temperature (Jolivet et al., 2023). The XRD results show that TiO_2_ is an amorphous structure when the temperature is 160°C, consistent with literature reports (Liu et al., 2017). Moreover, the TiO_2_ of different cycles did not show anatase titanium dioxide, which indicates that the crystalline form is not related to the number of cycles but to the working temperature. Anatase TiO_2_ may be toxic to osteoblasts (Bernier et al., 2012). So the amorphous TiO_2_ films in this study can avoid the toxicity problem. Furthermore, different cycle counts of ALD have been reported in the literature to have an effect on the corrosion resistance of zirconia films (Yang et al., 2017), but there is little literature comparing the friction and corrosion resistance of TiO_2_ deposited on the surface of titanium implants with different ALD cycles. This study was therefore conducted with three gradients of cycle numbers to investigate the relationship between cycle numbers and friction and corrosion resistance.

In this study, we assessed how different ALD cycles affect titanium implant performance, specifically focusing on species release and friction corrosion resistance. We simulated intraoral conditions with artificial saliva at 37°C. We found that the TiO_2_ coating initially experienced wear and corrosion, but it effectively protected the underlying titanium substrate. Thicker TiO_2_ coatings showed greater resistance to both wear and corrosion, providing better protection for the titanium implant.

ICP analysis of the immersion solution confirmed reduced ion levels, with the 500ALD-Ti group having significantly lower titanium ion concentrations compared to other groups. This aligns with our friction and corrosion results, confirming that the titanium dioxide film effectively limits the release of titanium species from the implant’s surface. Overall, increasing the number of ALD cycles enhances friction corrosion resistance and reduces titanium species release.

Despite the small thickness of TiO_2_ overcoating layers, they significantly contributed to the corrosion behavior of CP-Ti. The smaller corrosion current (Icorr) values of the coated samples obtained by fitting the dynamic potential polarization curves using Tafel curves indicate that the coatings effectively reduced the corrosion rate of Ti and protected its surface from electrochemical reactions. And the 500-cycle TiO_2_ films showed the smallest Icorr compared to the other two sets of coatings, a trend consistent with that reported in the literature (Kania et al., 2021). Moreover, Nyquist plots of impedance spectra showed that the experimental group had a larger semicircle diameter than the control group, while the 500-cycle group had the largest, which suggested that the films improved the insulating properties of the surface. In other words, the ALD films are better capacitors with a higher protection level than pure titanium. This may be due to the fact that the standard electrode potential of TiO_2_ is the same as that of titanium, which slows down the oxidation rate. This paper used three different methods to evaluate the corrosion resistance: dynamic polarization curves, EIS spectroscopy and immersion experiments. The results of the three methods are basically consistent. By increasing the number of ALD cycles of the TiO_2_ film, the corrosion resistance of the titanium surface can be significantly improved, the corrosion rate reduced, the corrosion area minimized, and the ions release reduced, providing an effective method for the corrosion protection of titanium materials in various applications.

It is worth mentioning that although the cell activity of the experimental group in the *in vitro* cell test was higher than that of the control group and increased with cycling, the cell activity of all four groups of samples was smaller than that of the blank group, and there was no statistical difference between the groups. Therefore, the experimental results can only show that TiO_2_ is not cytotoxic, but cannot prove that the coating can increase cellular activity. This is inconsistent with the report of Liu et al. on the ability of TiO_2_ to increase cytocompatibility (Liu et al., 2017), which may be due to the fact that the number of cycles in the literature is 2500, while the number of cycles in this experiment is less, and the uncoated pure titanium can also show high biocompatibility and stimulate cell differentiation in the osteogenic direction, so the difference between the experimental group and the control group is smaller.

While Atomic Layer Deposition (ALD) technology demonstrates significant advantages in surface modification, it still encounters challenges in large-scale manufacturing. The layer-by-layer deposition process of ALD results in a relatively slow preparation speed, as each layer needs to go through a complete cycle. This limitation hampers the technology compared to other surface modification techniques. To address this issue, optimizing the reaction conditions of ALD to enhance the efficiency of each cycle and consequently increase the preparation speed could be considered. Another significant challenge is reducing the cost associated with ALD in manufacturing. The ALD process requires substantial energy for heating and vacuum, making optimization in throughput crucial (Zhuiykov et al., 2017). Adjusting the reactor to maximize precursor utilization and reducing precursor costs is a potential solution. Additionally, exploring more economical and readily available precursor materials can effectively alleviate cost pressures.

From the comprehensive experimental results, it is worth affirming that the ALD-deposited TiO_2_ coating can both enhance the frictional corrosion resistance of pure titanium and reduce the release of titanium species. In this experiment, three sets of gradient cycle times were set, so it is presumed that the increase of ALD cycle numbers has positively affects the reduction of species release. However, no conclusion has been reached on the optimal number of cycles for the friction resistance and corrosion performance; therefore, the optimal number of cycles for the friction resistance and corrosion performance will be further investigated. Furthermore, the relationship between the biocompatibility of ALD-TiO_2_ films and their resistance to frictional corrosion remains uncertain. To address this uncertainty, we plan to conduct additional biocompatibility experiments in subsequent studies, examining the biological performance of TiO_2_ films under different frictional corrosion conditions. Through these experiments, we aim to determine the optimal number of TiO_2_ film cycles to achieve the best performance in various aspects.

## 5 Conclusion

In this study, TiO_2_ films were prepared on the titanium surface by the ALD technique, which significantly improved the friction corrosion resistance of the material and thus reduced the release of titanium species. Three different cycle designs demonstrated that the increase in the number of ALD cycles significantly impacted the morphology and properties of the TiO_2_ films, significantly improving the friction corrosion resistance of the TiO_2_ films and reducing release of titanium species. This provides important guidance for designing and preparing titanium implants with more resistance to friction and corrosion. Future studies can further explore optimizing ALD parameters and selecting coating materials to achieve better friction corrosion resistance and biocompatibility.

## Data Availability

The original contributions presented in the study are included in the article/Supplementary material, further inquiries can be directed to the corresponding authors.
